# Identifying type 2 diabetes risk classification systems and recommendations for review of podiatric care in an Australian Aboriginal health clinic

**DOI:** 10.1186/s13047-015-0089-2

**Published:** 2015-07-30

**Authors:** Lauren Blatchford, Pam Morey, Ruth McConigley

**Affiliations:** Albury-Wodonga Aboriginal Health Service, 644 Daniel St, Glenroy, NSW 2640 Australia; WoundsWest, Curtin University, GPO U 1987, Perth, WA 6845 Australia; School of Nursing and Midwifery, Curtin University, GPO U 1987, Perth, WA 6845 Australia

**Keywords:** *Indigenous health*, *Australia*, *Podiatry*, *Rural health*, *University of Texas*

## Abstract

**Background:**

There are several risk classification systems developed to facilitate diabetic foot assessments and prioritise diabetes patients for foot prevention services according to risk factors. Utilisation of both The University of Texas Diabetic Foot Risk Classification System (UTDFRCS) and The National Evidence-Based Guideline on Prevention, Identification and Management of Foot Complications in Diabetes (Part of the Guidelines on Management of Type 2 Diabetes), allows guidance for the podiatrist in terms of review timeframes for future assessments and treatment. The aim of this clinical audit was to classify Aboriginal type 2 diabetes subjects’ risk status according to UTDFRCS and identify if evidence based standards are being met for podiatry services at the Albury-Wodonga Aboriginal Health Service in New South Wales, Australia.

**Methods:**

A retrospective clinical audit over a twenty six month period was undertaken at the Albury-Wodonga Aboriginal Health Service, New South Wales. This is a primary health care facility that started podiatry services in August 2011. The primary variables of interest were the UTDFRCS for each subject and whether those participants met or did not meet the National Evidence-Based Guideline for review appointment timeframes. Other variables of interest include age, gender, duration of diabetes, occasions of visits and cancelled and failure to attend appointments to the podiatry service over the data collection period.

**Results:**

There was excellent overall adherence (94 %) of this sample population (*n* = 729) to the National Evidence-Based Guideline for podiatric review timeframes according to their risk status. Males were reported to be less likely to comply with the review timeframes compared to women. There was no association between risk status and age (*OR* = 1.04, *p* = 0.11), duration of diabetes (*OR* = 1.03, *p* = 0.71) or gender (*OR* = 0.77, *p* = 0.67).

**Conclusions:**

Regular foot examinations aid in stratifying patients according to risk status, guiding podiatry interventions to reduce the likelihood of ulceration and amputation. This primary health care setting has achieved podiatric evidence based standards for Aboriginal people with type 2 diabetes, demonstrated by acceptable timeframes for review appointments.

## Background

The prevalence of type 2 diabetes is growing in Australia [1], and for the Australian Aboriginal population it is reported as three times more common than in the non-Aboriginal population [2]. Diabetic foot related complications are the most severe and frequent complications of diabetes [3], including amputation of all or part of a lower extremity [4]. In order to prevent diabetes foot complications, evidenced-based risk assessment and diligent follow-up are crucial; however less than half of Australians with diabetes have regular foot examinations [5]. It is important to ensure that risk screening is undertaken in line with evidence-based guidelines to identify individual risk for complications and institute preventative care.

There are several risk classification systems developed to facilitate diabetic foot assessment and prioritise diabetes patients for foot prevention services according to risk factors, such as: previous foot ulceration, peripheral neuropathy, ill-fitting footwear, and socio-economic disadvantage [6, 7]. Classification systems can create confusion among health practitioners as to which should be adopted into practice [6]. The International Working Group on the Diabetic Foot (IWGDF) [4], the Diabetic foot Assessment of Risk Tool (DART) screening form [8], and the University of Texas Diabetic Foot Risk Classification System (UTDFRCS) [9], are risk classification systems. The IWGDF has been reported to undervalue the impact of peripheral arterial disease and history of amputation [6]. The UTDFRCS has been stated as not one of the simplest classification systems [10]. Currently the DART screening form is the only risk classification that is specifically tailored to Aboriginal populations; however there is little evidence available in the literature supporting its use [11]. The number of Aboriginal people having a stay in hospital due to diabetic foot ulcers or lower limb amputation is higher than non-Aboriginal people, and evidence suggests that risk screening and early intervention can reduce these numbers [11]. Identifying those Aboriginal people at high risk of developing diabetic foot complications is crucial in reducing the number of diabetic foot complications, and is supported with the use of specifically tailored risk classification systems [11].

The UTDFRCS (Table 1) has been shown to be a reliable and valid tool for predicting future foot-health outcomes for people with diabetes [9, 10]. The UTDFRCS consists of two parts; the first part stratifies patients into risk groups for ulceration, and the second stratifies patients with an existing ulceration into risk groups for amputation [10]. The National Evidence-Based Guideline on Prevention, Identification and Management of Foot Complications in Diabetes (Part of the Guidelines on Management of Type 2 Diabetes) [7], provides the gold standard of care for Aboriginal people with type 2 diabetes, and recommends follow up on a regular basis; however this evidence based guideline fails to categorise this population according to the stated risk factors, and automatically labels Aboriginal patients as high risk. Adoption of the National Evidence Based Guidelines stratifies the person as low, intermediate or high risk, and can then be linked to the risk status identified by the UTDFRCS [9], guiding the podiatrist for review timeframes of future assessment and treatment (Table 2). Through application of both the UTDFRCS and the National Evidence Based Guidelines, it is predicted that management can be tailored according to identified risk status and therefore reduce the number of diabetic foot complications in Aboriginal populations.

**Table 1:** The University of Texas Diabetic Foot Risk Classification System (adapted from [14])

**Category 0: Minimal Pathology**	**Category 1: Insensate Foot**	**Category 2: Insensate Foot with Deformity**	**Category 3: Demonstrated Pathology**
**NO Neuropathy**	**Neuropathy NO Deformity**	**Neuropathy with Deformity**	**History of Pathology**
• Patient diagnosed with diabetes mellitus.	• Patient diagnosed with diabetes mellitus.	• Patient diagnosed with diabetes mellitus.	• Patient diagnosed with diabetes mellitus.
• Protective sensation intact (Semmes-Weinstein 10g monofilament detectable).	• Protective sensation absent (Semmes-Weinstein 10g monofilament NOT detectable).	• Protective sensation absent.	• Protective sensation.
• Ankle brachial index of >0.8 and toe systolic pressure of >45mmHg.	• Ankle brachial index of >0.8 and toe systolic pressure of >45mmHg.	• Ankle brachial index of >0.8 and toe systolic pressure of >45mmHg.	Ankle brachial index of >0.8 and toe systolic pressure of >45mmHg.
• Foot deformity may be present.	• No history of ulceration.	• No history of ulceration.	• History of neuropathic ulceration.
• No history of ulceration.	No history of Charcot’s joint.	• No history of Charcot’s joint.	• History of Charcot’s joint.
	No foot deformity.	• Foot deformity present.	• Foot deformity present.
**Category 4A: Neuropathic Ulcer**	**Category 4B: Acute Charcot’s Joint**	**Category 5: Infected Diabetic Foot**	**Category 6: Dysvascular**
**Neuropathic Wound**	**Acute Charcot’s Joint**	**Infected Diabetic Foot**	**Foot Ischaemic Limb**
• Patient diagnosed with diabetes mellitus.	• Patient diagnosed with diabetes mellitus.	• Patient diagnosed with diabetes mellitus.	• Patient diagnosed with diabetes mellitus.
• Protective sensation may or may not be intact.	• Protective sensation absent.	• Protective sensation may or may not be intact.	• Protective sensation may or may not be intact.
• Ankle brachial index of >0.8 and toe systolic pressure of >45mmHg.	• Ankle brachial index of >0.8 and toe systolic pressure of >45mmHg.	• Infected wound.	• Ankle brachial index of <0.8 and toe systolic pressure of <45mmHg.
• Foot deformity normally present.	• Non-infected neuropathic ulceration may be present.	• Charcot’s joint may be present.	• Ulceration may be present.
• Non-infected neuropathic ulceration.	• Charcot’s joint present.		
• No Charcot’s joint present.

**Table 2:** Comparison of the University of Texas Diabetic Foot Risk Classification [9] and National Evidence Based Guidelines for risk status [7]

University of Texas risk category	University of Texas risk definition	National Evidence Based Guidelines risk status	National Evidence Based Guidelines frequency of foot examination
0	No Neuropathy	“low risk”- people with no risk factors and no previous history of foot ulcer/amputation	Annually
1	Neuropathy, no deformity	“intermediate risk”- people with one risk factor (neuropathy, peripheral arterial disease or foot deformity) and no previous history of foot ulcer/amputation	Every 3 - 6 months
2	Neuropathy with deformity	“high risk” - people with two or more risk factors (neuropathy, peripheral arterial disease or foot deformity) and/or a previous history of foot ulcer/amputation	Every 3 - 6 months
3	History of pathology		
4a	Neuropathic wound		
4b	Acute Charcot’s joint		
5	Infected diabetic foot		
6	Ischaemic limb		

The main goal of the clinical audit was to identify current standards of assessment and risk identification, and adherence to the implementation of best practice [12]. The aim of this study was to explore the occasions of visits to podiatry services at the Albury-Wodonga Aboriginal Health Service (AWAHS), and compare the results to evidence-based practice on suggestions for review timeframes according to the patient’s risk status. The audit aimed to identify whether evidence-based standards were being met for podiatry services at the AWAHS in reference to diabetes foot assessments. Albury is a major regional city located on the border of New South Wales and Victoria, with an estimated population at 2014 of 51,082, of which the Aboriginal population accounts for 1.6 % [13]. AWAHS services the Aboriginal community of Albury, Wodonga and the surrounding smaller towns. Services provided include medical, nursing, allied health, social and emotional wellbeing, dental, and health promotion. Podiatry services are offered two days a week by one podiatry practitioner.

## Methods

A retrospective clinical audit was conducted at AWAHS, to determine subject’s foot risk status with type 2 diabetes using the UTDFRCS [9], and comparing the occasions of visits against evidence based guidelines [7] review timeframes. Podiatry services were first commenced at this primary health care setting in August 2011; prior to this no Aboriginal podiatric service was available in this area. At the commencement of this service, the UTDFRCS was adopted to categorise Aboriginal diabetes patients’ risk for future foot related complications. The clinical information gathered and recorded at the first consultation to determine the risk status included: a diagnosis of type 2 diabetes, assessment of protective sensation using a 10 g monofilament, presence or absence of foot pulses on palpation, an Ankle-Brachial Pressure Index (ABPI), the presence of deformity, and current or prior history of ulceration and/or Charcot’s joint (Table 1). These determinants were then reviewed at each subsequent visit. The clinical information was collected by one podiatry practitioner throughout the data collection period. A standardised clinical assessment protocol was used and included the absence or presence of pedal pulses, the recommendation of undertaking an ABPI, and a loss of protective sensation recorded as absent if two out of three sites are not detected with the 10 g monofilament [3, 7].

The inclusion criteria for the sample were: Aboriginal or Torres Strait Islanders with a diagnosis of type 2 diabetes, aged 18 years and older, who had accessed AWAHS podiatry services after 1st January 2012. Subjects were excluded if they were employed at AWAHS, or if they had a diagnosis of Type 1 or gestational diabetes. A purposive sample was developed from obtaining an overall number (*n* = 729) of patients accessing podiatry services from 1st January 2012 to 6th March 2014, a period of 26 months. This number was generated through the analysis of appointments in the ZedMed computer based patient administration system utilised at AWAHS. The sample size (*n* = 70) was determined in accordance with the inclusion criteria and manual review of each patient’s medical history and background information recorded in the ZedMed computer based clinical notes. The occasions of visits were also retrieved manually and were identified by the number of appointments for each patient, collated from the appointment book in the ZedMed program.

Data was obtained from the patient’s clinical records and podiatry clinical notes. The primary variable of interest was the UTDFRCS [9], determined for each patient on the initial podiatry visit, and the incidence of new diabetes-related foot complications documented during subsequent visits, updating the patients’ individual risk status. Secondary variables included: the number of visits; cancelled and failed to attend appointments to the podiatry service over the 26 month period; and other basic demographic and medical variables that included age, gender, and duration of diabetes (years).

### Ethical considerations

Ethics approval was obtained from the Curtin University Human Research Ethics Committee (HR 06/2014) and the Aboriginal Health and Medical Research Council of New South Wales (977/13). A key ethical consideration was the Aboriginal population being studied, and acceptance for the project was gained from Aboriginal elders within the community and the Aboriginal Corporation Health Service. The data was de-identified to protect privacy and confidentiality particularly with the small sample size.

### Statistical analysis

Participant data is reported using descriptive statistics for age, gender, duration of type 2 diabetes, occasions of visits to podiatry, cancelled and failed to attend appointments, and foot risk status using the UTDFRCS. Chi-square was used to analyse the influence of gender on the subject’s attendance for those that met the evidence based review timeframes. Ordinal Regression was used to assess the effect of each independent variable (age, duration of diabetes, and gender) on risk status in general. The Mann–Whitney *U* Test was used to analyse whether a relationship existed between two groups (those participants that met the evidence based requirements of review timeframes and those that didn’t meet the requirements), with the variables of UTDFRCS, age and duration of diabetes.

## Results

### Demographic data

**Fig. 1:**
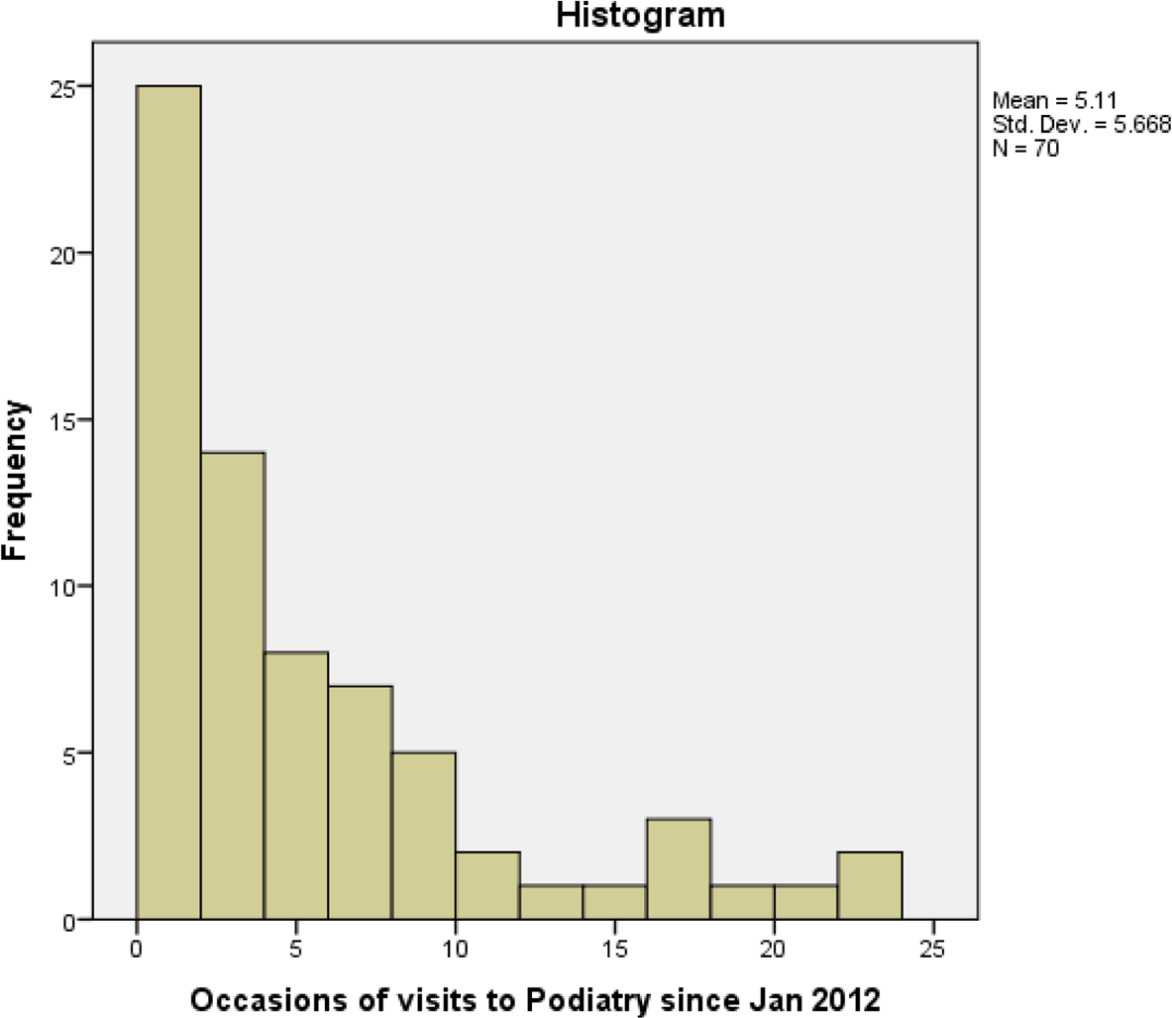
Occasions of visits to podiatry services at The Albury-Wodonga Aboriginal Health Service during a 26 month period

Data was collected from 70 Aboriginal patients with type 2 diabetes and participant data can be found in Table 3. The participants had a mean age of 55.43 years (range of 21 to 85 years), with a preponderance of females, and a mean duration of type 2 diabetes greater than five years (range of 1 to 16 years). Seventy eight percent of the sample had a UTDFRCS of ‘no neuropathy’ (Table 4). Just over 35 % of patients accessed podiatry services only once during the 26 month period of analysis, whilst just over 14 % accessed the service twice (Fig. 1). The remainder of the participants attended podiatry services between 3 and 23 times during the 26 month period of analysis. There was a high percentage (70 %) of participants that attended their podiatry appointments, with a small percentage (4.3 %) of participants that cancelled or failed to attend their appointment on two or more occasions during the data collection period.

**Table 3:** Participant data

Variable	Total (n=70)
Gender	
Female (%)	64.3
Male (%)	35.7
Age (years)	55.43 (mean)
Duration of diabetes (years)	5.74 (mean)

**Table 4:** Number of subjects in each University of Texas Diabetic Foot Risk Classification System

Category	Classification	Frequency	Percent
0	No neuropathy	55	78.6
1	Neuropathy, no deformity	9	12.9
2	Neuropathy, with deformity	2	2.9
3	History of pathology	0	0
4a	Neuropathic wound	2	2.9
4b	Acute Charcot’s joint	0	0
5	Infected diabetic foot	2	2.9
6	Ischaemic limb	0	0

### Sample

Four of the 70 subjects did not meet the required review timeframes as stated by evidence based standards [7] (Table 5). There was no statistically significant difference between genders for patients that met the evidence based requirements of review timeframes (*F* = 0.127, *p* = 0.91) and those who did not. Ordinal logistic regression showed that risk status was not significantly related to age (*OR* = 1.04, *p* = 0.11), duration of diabetes (*OR* = 1.03, *p* = 0.71) or gender (*OR* = 0.77, *p* = 0.67). The patients that did not meet the evidence based review timeframes had a higher risk status compared to those that did meet the review timeframes and this was noted to be a risk category of 1 or higher using the UTDFRCS (*U* = 11.5, *p* = 0.03). No difference existed between age and duration of diabetes, and subjects that met or did not meet the review timeframe requirements (*U* = 106, *p* = 0.533 and *U* = 125, *p* = 0.874 respectively).

**Table 5:** Participants that did not meet the evidence based requirements for review timeframes according to risk status

Gender	Age (years)	Duration of Diabetes (years)	University of Texas Risk Status	Occasions of visits to podiatry^a^
Male	57	9	4	1
Male	45	6	2	1
Male	68	4	1	1
Female	64	2	6	4

^a^During the 26 month data collection period

## Discussion

The design used in this study was a retrospective clinical audit, where recording of socio-demographic and foot-health variables, amongst an Aboriginal population with type 2 diabetes, attending an Aboriginal Community Controlled Health Service (ACCHS), in regional Australia was undertaken. A standardised clinical approach to assessment, diagnosis and management of the care of people with diabetes had been implemented since podiatry services at AWAHS began in 2011. This allowed for accurate data to be recorded with the implementation of the UTDFRCS [9]. The first part of the classification stratifies patients into risk groups for ulceration, and the second stratifies patients with an existing ulceration into risk groups for amputation [10]. This classification system is a logical and validated tool that has been shown to ensure efficient use of podiatric services [10]. Many studies have been developed that propose a range of classification systems that can identify patients at risk of foot ulceration, creating confusion among health practitioners as to which should be adopted into practice [6]. When the International Working Group on the Diabetic Foot [4] is compared to the UTDFRCS, it has been shown to undervalue the impact of peripheral arterial occlusive disease and history of amputation as risk factors for ulceration, whilst the DART [8] system lacks evidence to support its use in the clinical environment.

An innovative aspect of the UTDFRCS adapted by the Department of Health Western Australia [9], is that it aligns with other guidelines for treatment and review, assisting the clinician to allocate resources, to determine the frequency of follow-up visits, and to document that foot screening is being performed on a regular basis [10]. However, this information is lacking in terms of Aboriginal health and their high risk status, and therefore requires the consideration of the National Evidence Based Guidelines [7].

The National Evidence Based Guidelines outline best practice based on rigorous systematic reviews, that state “until adequately assessed, all Aboriginal and Torres Strait Islander people with diabetes are considered to be at high risk of developing foot complications and therefore will require foot checks at every clinical encounter and active follow-up” [7]. Once assessed, this population can then be stratified as low, intermediate or high risk, and can be linked to the risk status identified by the UTDFRCS [9]. This evidence based guideline suggests those that are low risk undertake a diabetes foot exam annually, whilst those that are intermediate or high risk, diabetes foot examinations should be undertaken every three to six months [7].

Implementation of the National Evidence Based Guidelines allows the person to be stratified, and can then be linked to the risk status identified by the UTDFRCS [9], guiding the podiatrist for review timeframes of future assessment and treatment.

Basic demographic and diabetes-related information gathered in this study further enhances the understanding of this high-risk population. There was an uneven proportion of males and females in the sample, with the majority being female. The distribution of ages for the subjects in the sample suggested that many were over the age of fifty years. This is consistent with other studies reviewing Aboriginal health that found females are more likely to access health services and were generally older [15–18]. Stout, Kipling and Stout [19], report that the lack of sufficient security and anonymity when using health services is experienced by male clients. Aboriginal women derive the greatest benefit from services and resources that are relevant to their cultural contexts [19], which could provide an understanding for the gender differences in terms of access to health care at AWAHS; however, consideration was not given to the population characteristics for the Albury-Wodonga region when compared to the clientele using the health service, and therefore this may not be a true representation of the Albury-Wodonga Aboriginal population accessing podiatry services.

The mean duration of diabetes observed in this study is consistent with a prior study in a remote Australian Aboriginal community that found a mean duration of diabetes of 5.6 years [15]. A high proportion of subjects were classified as having no neuropathy and were considered low risk according to the National Evidence Based Guideline [7]. This result correlates with the literature that analysed diabetes care and complications in a similar health care setting. The authors found numbers as low as seven and as high as twenty four percent of subjects respectively, were diagnosed with neuropathy [15, 20]. One study analysing Aboriginal type 2 diabetes complications among Canadians in a community based setting, did report over 46 % of participants were diagnosed with neuropathy; however the clinical test used to diagnose neuropathy was not consistent with that of the UTDFRCS [18].

This study concluded a high number of participants accessed podiatry services on a one off basis. This could be related to the lack of information, limited literacy or poor understanding of health information that frequently led to non-compliance [21]; however, a recent Cochrane review suggests that patient education for the prevention of diabetes-related foot complications is yet to be proven to be effective, with education possibly having positive outcomes on foot-care behaviours in the short term only, with a yet unknown effect on long term foot-health outcomes [22]. It has also been suggested that Aboriginal people rely on their community for support in their health management and are consistently likely to seek health information from family and friends before seeking health care from health services [23], including podiatry.

The recent literature suggests males, those subjects that are older, and the longer the duration of diabetes, are more likely to have a higher risk for diabetes related foot complications [20, 24], which was evident in this study. During the development of the University of Texas risk classification system, Lavery and colleagues found that being male and having experienced a longer duration of diabetes were associated with higher risk diabetes-related foot complications [5]. This study also concluded males were more likely to not comply with review timeframes when compared to women, and were associated with one occasion of visit during the twenty six month data collection period, a higher risk status and a longer duration of diabetes.

Zhang and colleagues [25], analysed Australian Aboriginal general practice encounters using the Bettering the Evaluation and Care of Health program. They identified that there was no difference between genders or age groups for Aboriginal people accessing health services for their type 2 diabetes care in terms of meeting recommended guidelines for follow up care [25]. This coincides with the results of this study which concluded no difference between gender and age of the ninety four percent of the sample that met the evidence based guidelines for review timeframes.

The high percentage of subjects that met the evidence based review timeframe requirements could be related to the strategy of the health service which is a community controlled organisation. Such organisations are the service of choice for Aboriginal people as they attempt to make their places and temporalities welcoming in culturally specific ways [23]. These results correlate with a recent study that explores how structuring of place and time influence Aboriginal patient experiences in health services [23]. The authors identified that if participants felt culturally safe within the health service environment, had enough time with the health care workers, and were able to have a ‘yarn’, the Aboriginal community would be more likely to return to the health service in future. Podiatry services at AWAHS allows for drop in appointments and provides transport services for people who do not have access to the clinic, allowing for flexible arrangements for the community to contact this particular service. The results of this study reflects the literature on ACCHS [21, 23] which contend that health services are more successful when utilising community engagement and fostering strong relationships in the community with health professionals. Ownership of the Albury-Wodonga health service is seen by the local Aboriginal community and is an ACCHS, which is required to achieve sustainable improvements in health behaviours, particularly in rural areas [21].

## Conclusion

Podiatrists are health professionals that provide diabetes patients with the appropriate screening, treatment and education on risk factors for foot complications, such as ulcerations and amputations. It is well known that the Aboriginal population have higher morbidity and mortality rates associated with diabetes foot related complications. Regular foot examinations alone have not decreased the incidence of ulceration and amputation in this population; however they do assist in stratifying patients according to risk status which guides podiatry interventions to assist in reducing the likelihood of ulceration, and subsequent amputation. This study illustrated that the majority of patients that visited podiatry services at the AWAHS met the evidence based guidelines for review timeframes, according to their UTDFRCS. The few that did not meet the review timeframe requirements, as set out by the guidelines, were male, had one occasion of visit during the twenty six month data collection period, a higher foot risk status and a longer duration of diabetes, which can be related to the cultural context of gender disparities in the Aboriginal culture.

### Limitations

The results of this study should be qualified in the light of limitations in the study design. This study was conducted in a single care setting with a small sample size. While the findings are relevant to this Aboriginal health service, they cannot be generalised to other ACCHSs. The ZedMed database has data collected commencing from 2005, and some subjects did not have a diabetes diagnosis date recorded that preceded this time. This has impacted on the results for the duration of diabetes in relation to risk factors and meeting the evidence based requirements of review timeframes. The very small number of participants that did not meet the evidence based requirements for review timeframes does not allow for meaningful statistical comparisons with those participants who met the requirements. Recommendations for future practice would include ensuring the correct documentation of the date of diabetes diagnosis. Further research is required that allows accurate information to be recorded, and especially of a qualitative design to ascertain the benefits and barriers of Aboriginal people accessing podiatry services as part of their diabetes care. This could assist understanding of the reasons for failure to access podiatry services after initial review.

## Abbreviations

UTDFRCS: University of Texas Diabetic Foot Risk Classification System
AWAHS: Albury-Wodonga Aboriginal Health Service
ACCHS: Aboriginal Community Controlled Health Service
IWGDF: International Working Group on the Diabetic Foot
DART: Diabetic foot Assessment of Risk Tool
